# Editorial: Enhancing learning through cognitive and social inclusion practices in education

**DOI:** 10.3389/fpsyg.2026.1938523

**Published:** 2026-08-13

**Authors:** Pedro Jesús Ruiz-Montero, María Luisa Santos-Pastor, Paula Batista

**Affiliations:** 1 Department of Physical Education and Sport, Faculty of Sport Sciences. University of Granada, Granada, Spain; 2 TEPAS Research Group HUM-1080, Department of Physical Education and Sport, Faculty of Sport Sciences, University of Granada, Granada, Spain; 3 Department of Physical Education, Sport and Human Movement, Autonomous University of Madrid, Madrid, Spain; 4 Faculty of Sport, University of Porto, Porto, Portugal; 5 Research Centre for Research, Education, Innovation and Intervention in Sport (CIFI2D), Porto, Portugal; 6 Centre for Research and Educational Intervention (CIIE), Porto, Portugal

**Keywords:** cognitive processes, diversity, educational practices, learning environments, pedagogical innovation

## 1 Introduction

Overall, the growing body of research in educational psychology highlights the need to understand learning as a multidimensional process in which cognitive, motivational, emotional, and social factors interact to shape educational outcomes (Immordino-Yang et al., 2019). Rather than being viewed solely as the acquisition of knowledge, learning is increasingly recognized as a socially situated process that depends on meaningful participation, supportive relationships, and equitable learning opportunities (Ainscow and Messiou, 2025; Biesta, 2022). Consequently, promoting cognitive and social inclusion has become a major priority for researchers, educators, and policy makers seeking to improve both educational quality and equity across educational systems (UNESCO, 2024). This evolving perspective has substantially enriched educational research while simultaneously revealing new theoretical and practical challenges.

Within this framework, increasing attention has been devoted to active and student-centered pedagogies capable of simultaneously promoting academic achievement, wellbeing, and inclusion. Educational approaches such as Service-Learning, collaborative learning, peer assessment, mentoring, digital learning environments, and other participatory methodologies have consistently demonstrated their potential for strengthening self-regulated learning, critical thinking, learner engagement, and social participation (Jackson-Summers et al., 2024). Contemporary educational research therefore suggests that cognitive development and social inclusion should not be considered independent educational goals, but rather mutually reinforcing dimensions of meaningful learning (Ainscow and Messiou, 2025). This perspective is consistent with socio-constructivist approaches and with growing evidence demonstrating that inclusive educational practices enhance both learning processes and students' opportunities for participation and success (OECD, 2023).

Beyond these pedagogical developments, contemporary education faces significant challenges arising from increasing cultural diversity, widening educational inequalities, and rapid technological transformation (Zhang et al., 2024). These developments require educational research to move beyond examining isolated psychological variables toward more comprehensive approaches capable of explaining how cognitive processes, socio-emotional development, pedagogical innovation, and contextual factors interact across diverse educational settings. Recent international reports similarly emphasize that inclusive education should be understood as a systemic process requiring coordinated action at classroom, institutional, and policy levels (Ainscow and Messiou, 2025; OECD, 2023; UNESCO, 2024).

Together, these developments reinforce the need to design learning environments that integrate cognitive engagement, social participation, and inclusive pedagogies as complementary dimensions of educational quality.

Against this background, the Research Topic Enhancing Learning through Cognitive and Social Inclusion Practices in Education seeks to advance understanding of how educational psychology can contribute to improving teaching and learning through cognitively and socially inclusive educational practices (Korthals Altes et al., 2024).

The contributions included in this Research Topic encompass empirical studies, intervention research, qualitative investigations, reviews, and conceptual contributions that collectively advance current knowledge in this field. Although the articles differ in methodology, educational level, and research context, they share a common focus on understanding how innovative pedagogical approaches can simultaneously foster cognitive development, social inclusion, learner wellbeing, and equitable educational participation. More specifically, the published studies examine how these educational approaches influence cognitive processes and learning outcomes, explore the psychological mechanisms underlying active learning methodologies—particularly among vulnerable populations—and identify educational practices capable of promoting equitable participation, academic success, and learner wellbeing across diverse educational contexts. Collectively, the Research Topic provides an interdisciplinary perspective on the dynamic relationships among cognition, inclusion, and educational practice while offering evidence to inform future research, educational innovation, and evidence-based educational policy (Ruiz-Montero et al., 2023).

## 2 Overview of the contributions

The Research Topic brings together a diverse body of research that reflects the growing international interest in cognitively and socially inclusive educational practices across different educational settings, populations, and methodological traditions. During the call for papers, which remained open between February 2025 and June 2026, a total of 82 manuscripts were submitted (121 authors), with 38 manuscripts successfully completing the review process and being accepted for publication, including one correction article. The resulting Research Topic is predominantly composed of empirical quantitative studies, with cross-sectional designs representing the largest methodological group. The Research Topic also includes a substantial number of intervention studies, mainly based on experimental and quasi-experimental designs, several qualitative and mixed-methods investigations that provide an in-depth understanding of educational processes, one meta-analysis, one narrative review, and one conceptual perspective article. Although a few studies incorporated longitudinal follow-up components, these were relatively limited in number. Geographically, the Research Topic is dominated by research conducted in China (*n* = 23; 62.2%), reflecting the growing prominence of research on cognitive and social inclusion within Asian educational contexts. Additional contributions were carried out in Spain (*n* = 3; 8.1%), Turkey (*n* = 3; 8.1%), Italy (*n* = 1; 2.7%), Indonesia (*n* = 1; 2.7%), Ukraine (*n* = 1; 2.7%), Hungary (*n* = 1; 2.7%), Libya (*n* = 1; 2.7%), and the United States (*n* = 1; 2.7%). Furthermore, two articles (5.4%)—a narrative review and a meta-analysis—synthesized evidence from multiple international contexts rather than focusing on a single country.

Regarding the participant profiles represented across the published studies, university populations clearly predominate, particularly students from Chinese higher education institutions. Nevertheless, the Research Topic also encompasses a broad spectrum of educational stakeholders, including children in early childhood, primary, and secondary education, teachers, families, older adults, and groups commonly associated with inclusive education, such as individuals with intellectual disabilities, Roma students, Hispanic children with learning difficulties, and international students. This diversity of participants broadens the scope of the Research Topic and reflects the multidimensional nature of cognitive and social inclusion across the educational lifespan and a wide range of educational contexts.

All accepted contributions can be organized into four predominant thematic areas, although some articles address more than one area due to the interdisciplinary nature of their research questions and findings. These areas are: (a) Cognitive Processes and Learning; (b) Motivation, Wellbeing, and Socio-emotional Development; (c) Educational Practices and Teaching Innovation; and (d) Inclusion, Diversity, and Educational Contexts. At the same time, these thematic areas are structured around four guiding questions that provide a coherent framework for organizing the published contributions: (a) Which processes enable learning and improve academic performance?; (b) Which personal and relational factors sustain engagement in learning?; (c) Which pedagogical practices, educational interventions, and teaching methodologies are implemented to create more effective learning conditions?; and (d) For whom, in which educational contexts, and under what forms of inequality does learning take place?

The first thematic area, Cognitive Processes and Learning, examines the psychological and cognitive mechanisms that enable knowledge acquisition, regulation, and application across different educational stages and disciplinary contexts. Representative topics include attention, memory, executive functions, reasoning, critical thinking, reading comprehension, disciplinary competence, self-regulated learning, metacognition, learning strategies, feedback processes, academic self-efficacy, and academic achievement.

The second area, Motivation, Wellbeing, and Socio-emotional Development, brings together research focusing on the emotional, motivational, relational, and identity-related factors that foster students' engagement, adjustment, and personal growth in educational settings. This area encompasses topics such as intrinsic motivation, autonomy, competence, relatedness, academic engagement, anxiety, resilience, psychological wellbeing, emotional regulation, professional identity, career calling, satisfaction, sense of belonging, social support, and socio-emotional competence.

The third area, Educational Practices and Teaching Innovation, includes studies devoted to the design, implementation, and evaluation of pedagogical approaches, instructional strategies, educational programs, and teaching resources intended to transform teaching and learning processes. The contributions within this area address innovative practices such as Service-Learning, flipped classrooms, peer assessment, collaborative learning, career guidance interventions, digital learning environments, educational uses of social media, shared book reading, neurodidactic programs, mentoring, and the design of learning tasks and feedback processes.

Finally, the fourth area, Inclusion, Diversity, and Educational Contexts, encompasses studies exploring how personal, social, cultural, linguistic, family, and institutional factors influence participation, educational opportunities, and equity across learning environments. Representative topics include disability, ethnicity, gender, migration, socioeconomic inequalities, minority students, linguistic diversity, ageism, discrimination, barriers to participation, school culture, classroom climate, family–school relationships, rural and urban educational contexts, and equitable participation in education.

### 2.1 Area 1—Cognitive processes and learning (10 articles)

At the earliest stages of development, Yi et al. found that educational interventions received before age three were associated with stronger cognitive development in later schooling. The effects varied according to socioeconomic and geographical conditions and were partly explained by gains in early literacy and writing skills, highlighting the importance of early learning experiences for subsequent academic success. Similarly, Zhumabayeva et al. showed that a neurodidactic program improved intelligence, attention, reasoning, and self-regulation among primary school pupils. Together, these studies emphasize that early educational experiences and neuroscience-informed interventions provide essential foundations for later cognitive development and academic achievement.

Moving to disciplinary learning, Zhu et al. found that students' epistemological beliefs influenced chemistry competence both directly and through critical thinking and learning approaches. Likewise, Cai et al. reported that supervisor support enhanced graduate students' critical thinking through increased academic passion and engagement. Taken together, these findings indicate that academic achievement depends not only on the acquisition of disciplinary knowledge but also on learners' beliefs about knowledge and their capacity to engage critically with learning.

A second group of studies emphasized the role of metacognition and self-regulation. Xue and Khalid found that monitoring, regulation, and self-efficacy positively predicted academic performance among distance learners. Similarly, Xu and Guénier demonstrated that self-regulation was a stronger predictor of translation competence than motivation, highlighting the importance of goal setting, self-monitoring, and strategic adaptation. The relevance of self-regulated learning was also evident in Wang et al., who found that ICT use supported self-regulation through information retrieval and social interaction. Collectively, these studies highlight self-regulated learning as a central mechanism through which learners actively construct knowledge across both face-to-face and digital learning environments.

Another line of research focused on learners' active engagement with feedback processes. Yang et al. identified attitudes, subjective norms, and perceived behavioral control as important predictors of students' engagement in self-feedback. Complementing these findings, Yang et al. showed that feedback self-efficacy mediated the relationship between self-feedback and academic achievement. These findings reinforce the growing recognition of feedback as a self-regulatory process through which students assume greater responsibility for monitoring and improving their own learning.

Finally, Orosco et al. demonstrated that comprehension strategy instruction improved mathematical achievement among Hispanic children with learning difficulties. These findings highlight the contribution of strategic processing, working memory, and comprehension skills to successful learning.

### 2.2 Area 2—Motivation, wellbeing, and socio-emotional development (11 articles)

The eleven articles in this area examine the personal and relational conditions that sustain students' engagement, adjustment, and development across educational contexts.

Several contributions highlighted the importance of vocational beliefs and professional identity as motivational resources. Li et al. found that proactive personality reduced career choice anxiety through lower intolerance of uncertainty and fewer decision-making difficulties.

Likewise, Wu et al. reported that career calling enhanced learning engagement among medical students through self-efficacy and achievement motivation.

These findings suggest that personal agency and vocational purpose play an important role in sustaining engagement during educational transitions.

A similar pattern emerged in teacher education. Guo and Tian found that professional identity and learning satisfaction were significant predictors of pre-service teachers' willingness to teach. Together, these studies suggest that personal agency, vocational purpose, and positive professional self-perceptions constitute key motivational resources that sustain engagement and commitment during educational and professional transitions.

The role of supportive relationships was emphasized in several studies. Yang and Chen showed that mentoring practices promoting autonomy, competence, and relatedness enhanced student teachers' motivation, engagement, and professional identity during internships.

Likewise, Debreli and Abdulqadir reported that feedback, classroom interaction, and autonomy-supportive teaching were among the strongest contributors to learner motivation and confidence in EFL classrooms.

The significance of social support extended beyond traditional classrooms. Yang et al. demonstrated that social support, self-regulated learning, and flow experiences strengthened engagement in self-directed e-learning.

Similarly, Gebre et al. found a positive association between teacher socio-emotional competence and student engagement, highlighting the importance of emotionally supportive learning environments.

Collectively, these findings highlight the central role of supportive interpersonal relationships in creating learning environments that foster motivation, confidence, and sustained educational engagement.

A third group of studies focused on wellbeing and socio-emotional resources. Rodrigues et al. found that a relaxation-based intervention improved emotional awareness and psychological wellbeing among university students. In a different educational stage, Schapira and Grazzani concluded that shared book reading promotes empathy, emotional understanding, and social competence in early childhood.

The importance of resilience was highlighted by Sánchez-Jiménez et al., who showed that Service-Learning strengthened pre-service teachers' adaptive capacity and tolerance of challenges. Finally, Peng et al. found that parental burnout negatively affected students' achievement through reduced academic self-efficacy, demonstrating that engagement in learning is influenced not only by educational contexts but also by family relationships and emotional support.

### 2.3 Area 3—Educational practices and teaching innovation (8 articles)

The eight articles included in this area examine pedagogical practices, educational interventions, and teaching methodologies designed to generate better learning conditions. Several contributions focused on the use of innovative methodologies and technologies. Uzun found that Instagram-based speaking tasks reduced speech anxiety and increased participation among international students learning Turkish, illustrating the potential of social media to support authentic language learning. Similarly, Ling and Jan reported that flipped classrooms promoted student-centered learning, autonomy, and pedagogical flexibility, although challenges related to technological and organizational demands remained. Together, these studies demonstrate that digital technologies can enrich learning when they are intentionally integrated into pedagogical designs that actively engage students rather than simply introducing new technological tools.

Other studies examined structured educational interventions. Wang and Madihie demonstrated that a program designed to develop mathematical resilience significantly improved students' capacity to cope with mathematical challenges. Likewise, Zhang et al. found that career planning education enhanced career adaptability and career decision-making self-efficacy, preparing students to manage increasingly complex educational and professional transitions. These findings highlight the value of educational programs that deliberately foster learners' adaptive capacities and equip them with competencies extending beyond immediate academic achievement.

Collaborative approaches also emerged as important pedagogical strategies. Cheng et al. showed that collaborative post-discussion assignments generated stronger learning outcomes than individual tasks, particularly when deeper conceptual understanding was required. Similarly, Yang et al. found that peer assessment improved academic performance, engagement, and self-regulated learning while fostering trust and critical reflection among students. Collectively, these studies reinforce the view that collaboration and shared responsibility for learning create opportunities for deeper cognitive processing and more active student participation.

The value of learning beyond conventional classrooms was highlighted by Wu, who emphasized the educational contribution of museum-based learning and the importance of teacher involvement in implementing such experiences. Finally, Asri et al. demonstrated that active participation, supportive communication, and positive group climates enhanced learning and student satisfaction, underlining the importance of relational and cultural dimensions within collaborative learning environments.

### 2.4 Area 4—Inclusion, diversity, and educational contexts (8 articles)

The eight articles in this area examine for whom, in which contexts, and under what conditions learning takes place, paying particular attention to participation, diversity, and educational inequalities.

Several studies focused on barriers to participation within educational contexts. Shi found that classroom silence among Chinese university students emerged through interactions between personal, contextual, and cultural factors that restricted participation. Similarly, Turan demonstrated that differences in school academic culture shaped reading achievement, illustrating how learning opportunities may vary across institutional environments. Together, these studies demonstrate that participation and academic success are influenced not only by individual characteristics but also by the cultural and organizational features of educational settings.

At the institutional level, Hu argued that increasingly diverse postgraduate populations require more inclusive and human-centered management practices capable of responding to varied academic and psychological needs. This contribution extends the discussion beyond classroom practices by emphasizing the role of institutional policies and organizational structures in promoting inclusive educational environments.

Other studies examined groups that commonly encounter barriers to participation. Aguado-Gómez et al. showed that an inclusive Service-Learning program promoted belonging, confidence, and meaningful learning among university students with and without intellectual disabilities. Likewise, Zaimoglu and Dagtaş found that critical pedagogy encouraged future teachers to engage with issues of equity, power, and social justice. Collectively, these findings illustrate how inclusive pedagogies can simultaneously strengthen educational participation and promote critical awareness of social inequalities.

The contribution of community-based and intergenerational experiences was explored by Ruiz-Montero et al., who demonstrated that intergenerational Service-Learning fostered empathy, cooperation, and positive attitudes toward aging among primary school students. Social inequality was particularly evident in the study by Moreira et al., which showed that school engagement acted as a protective factor for Roma students while also revealing the continuing influence of discrimination and structural barriers on educational opportunities. Finally, Ma et al. examined gender-related barriers to physical activity participation. Their findings suggest that supportive and gender-responsive teaching strategies can help address social and cultural constraints affecting female university students' engagement in sport and physical activity.

## 3 Emerging themes and future directions

Collectively, the contributions included in this Research Topic provide a comprehensive overview of how cognitive and social inclusion can enhance learning across diverse educational contexts. Despite their methodological and contextual diversity, four overarching themes emerge. First, the findings consistently support a multidimensional conception of learning, demonstrating that academic achievement depends not only on cognitive and metacognitive processes but also on motivational, emotional, relational, and contextual factors. Second, the widespread adoption of active, student-centered pedagogies, including Service-Learning, collaborative learning, peer assessment, flipped classrooms, digital learning environments, and mentoring programs, reflects a shift toward educational approaches that position learners as active agents in the construction of knowledge. Third, the contributions reinforce the view that inclusion extends beyond access to education by requiring learning environments that promote participation, belonging, equity, and meaningful engagement. Finally, digital technologies emerge not as educational goals in themselves but as pedagogical resources whose value depends on their capacity to foster cognitive engagement, self-regulation, collaboration, and inclusion.

Despite these advances, this Research Topic highlights several important knowledge gaps. Most of the studies were conducted in higher education settings, particularly among Chinese university students, whereas research focusing on early childhood, primary, secondary, vocational, and adult education remains comparatively underexplored. Likewise, although the Research Topic includes several populations traditionally associated with inclusive education—such as individuals with intellectual disabilities, children with learning difficulties, and older adults—many other groups experiencing educational vulnerability continue to be underrepresented. Similarly, while studies focusing on individual psychological variables are abundant, fewer investigations adopt an ecological perspective capable of simultaneously examining the interactions among students, teachers, families, schools, and communities. Finally, cross-national comparative research remains scarce, thereby limiting opportunities to understand how inclusive educational practices operate across different educational systems and sociocultural contexts.

Methodologically, cross-sectional quantitative designs predominate, providing valuable evidence on relationships among variables but offering limited insight into causal mechanisms and developmental change over time. Although intervention studies, qualitative research, mixed-methods investigations, a meta-analysis, and a narrative review broaden the methodological landscape, longitudinal evidence remains scarce and generally involves short follow-up periods. Future research would therefore benefit from greater methodological diversity, including longitudinal, multicenter, implementation, and international comparative studies, alongside mixed-methods approaches capable of integrating quantitative and qualitative evidence.

Taken together, these findings suggest several priorities for future research. Expanding the geographical diversity of the educational contexts under investigation, increasing the representation of underexplored educational stages and populations, and examining inclusion from more systemic and multilevel perspectives should be considered key priorities. Furthermore, greater attention should be devoted to investigating the effectiveness and scalability of innovative educational interventions, particularly those integrating digital technologies, artificial intelligence (AI), collaborative learning, and community-based pedagogical approaches. Finally, future studies should pay greater attention to the complex interactions among cognitive, socio-emotional, pedagogical, and contextual variables by adopting interdisciplinary theoretical frameworks capable of capturing the multidimensional nature of inclusive educational processes. Advancing research in these directions will strengthen the evidence base while supporting educational policies and practices aimed at promoting more equitable, meaningful, and sustainable learning opportunities.

## 4 Conclusions

Taken together, the contributions included in this Research Topic collectively demonstrate that learning is a multidimensional process shaped by the interaction of cognitive, motivational, socio-emotional, pedagogical, and contextual factors. Although the Research Topic is predominantly represented by studies conducted in higher education and within the Chinese context, it offers valuable evidence across a range of educational settings, participant groups, and methodological approaches. Across the four thematic areas identified -Cognitive Processes and Learning; Motivation, Wellbeing, and Socio-emotional Development; Educational Practices and Teaching Innovation; and Inclusion, Diversity, and Educational Contexts—a consistent message emerges: meaningful learning is strengthened when cognitive development is accompanied by opportunities for participation, support, inclusion, and belonging.

Whether through self-regulated learning, collaborative pedagogies, mentoring, Service-Learning, digital learning environments, or interventions designed to support diverse learners, the studies consistently demonstrate that educational quality and equity are mutually reinforcing rather than competing goals. Collectively, this Research Topic provides evidence that can inform future research, educational innovation, and policy development while reinforcing the importance of designing learning environments that enable all learners not only to achieve academically but also to participate meaningfully, develop a sense of belonging, and flourish.
